# Discussions in the Mississippi Valley Dental Society

**Published:** 1872-04

**Authors:** 


### DENTAL DISCUSSIONS.

### Discussions in the Mississippi Valley Dental Society.—28th Annual Meeting.

The following discussions took place at the 28th Annual Meeting of the Mississippi Valley Dental Society, held in the Dental College, Cincinnati, commencing March 6th, 1872, and continuing three days:

SUBJECT 1ST.—"What are the legitimate requirements and expectations of the dentist in view of the necessities that exist, and the advantages available?"

Dr. H. McCollum, of Augusta, Ky., opened the discussion of this proposition with the following paper:

SUBJECT 1ST.—What are the legitimate requirements and expectations of the dentist in view of the necessities that exist, and the advantages available?

As in all other avocations the requirements of the dentist must be directed or addressed to himself; his expectations towards others.

The requirements of the dentist demand, first that all due diligence be given to the acquirement of the instruction and training necessary to the highest and fullest qualification for the duties of his chosen avocation.

2D.—That he sacrifice whatever of personal ease, previous habit, or constitutional defect, or infirmity stands opposed to an entire qualification for the correct and successful performance of the duties of a dentist.

3D.—That he divest himself as far as possible of all contemptible, pettifogging, Jew-pedlar tricks of trade that too often sully the splendor or mar the beauty of otherwise honorable occupations, and that he maintain in all respects before all the habitants of heaven and earth a moral, social, and personal character above reproach.

Having thus met the legitimate requirements he is entitled to expect first the unreserved confidence of his patrons. No one can practice dentistry with credit to himself or benefit to others on any other basis.

2d.—He is entitled to expect the distinguished regards of his professional brethren. This proposition needs no argument or elaboration.

3d.—He is, moreover, entitled to expect a comfortable place in the general estimation of society at large, in common with all other avocations necessary to the well being of society.

4th.—He is entitled to expect an adequate pecuniary support.

I am not among those who estimate a man's social or moral worth by the amount of pecuniary compensation that he claims or receives, but the necessity for material support brings all men on a common level. The "best and bravest" among us can not afford to labor for nothing, and in dentistry, as in all beneficent enterprises, "the laborer is worthy of his hire." These are not all, but some of the most obvious thoughts that present themselves in response to the above question.

Many of the obstacles that impede the progress of dentistry arise out of the lamentable fact that so many heedlessly and recklessly push themselves into the profession and incontinently demand, while they are destitute of any legitimate right to expect, the honors and emoluments that are justly accorded to such as prove themselves worthy of the position to which they aspire.

Dr. J. Taft observed on behalf of the Executive Committee that in preparing the subjects for discussion they aimed to get out of the old ruts and give a different direction to the thought and expression of the Society. He thought it would not be possible to bring every mind up to the same standard. There appeared to be a false view in regard to this. Many think, for instance, that what one man does another can also do. This is not so, for each is not qualified as the other. Men are neither equally endowed nor gifted with capacities. There is a strong individuality inwrought with each man's being. Then, again, circumstances and opportunities are not distributed alike. The lives of some men abound with both; while, again, there are men who live out their alotted time in total barrenness of either or both. Locality, condition, society, the very geography of a place not only influences but often determines a man's achievements. The teeth of a people are all more or less affected by various controlling circumstances, and they must be taken into account by the dentist. The question then arises, "What is his work?" It is to ameliorate the condition of mankind, to ward off disease, ana to conserve the well-being of the organs to which he gives attention. The point here is to recognize what we require.

A patient calls for a filling, and expects it not only to last forever but somehow or other gets it into his head that because he has been to the dentist, that that is sufficient, and never after troubles himself about his teeth till pain brings him to his senses. Now he should be taught not to expect so much; not to place his expections too high. But, on the other hand, there is a class who place too low an estimate on the dentist and what he can do for them, and never feel called to go near the dentist until brought by suffering, or impelled by the example of some better enlightened neighbor. Now, as these both misapprehend and misappreciate the function of the dentist, it remains for him to teach them better. The people must be educated up to know and understand what the dentist can do for their comfort, health, and improvement.

With regard to what is to be expected of the dentist. It is reasonable to expect him to preserve the teeth permanently. That should be the standard expectation of the dentist. It is in his province to save the teeth already diseased, and to restore the diseased organs to a proper performance of their functions, but it will require skill, care, and perseverance to do this. Attention must go farther than the mouth. It should be required of the dentist to understand the diseases operating on the mouth. But it may be asked, "What if he can not ameliorate them?" Why, then he still possesses the advantage of an enlightened knowledge. Again, it may be asked, "Is it really expected that the dentist should possess this knowledge?" Most assuredly it is, and it is only when he has attained it and can act upon it that he becomes strong, efficient, and valuable.

Dr. Geo. Watt observed that it would be well to remember that, as a profession, dentists practiced on a race that, as a rule, is not healthy. He did not believe there was such a thing as perfect health.

He would look at the question from the standpoint of the dentist. The dentist must expect failures and plenty of them. He must be patient, however, and not manifest his chagrin. Inasmuch as the mind exercises a controlling influence over the body and has a diseased machine to act through, he must expect to meet very unreasonable people. He must act then without regard to this as though it were not operating. A man who goes into any department of medical science must expect to meet unreasonable people, but if he can not bear them, especially if he is a dentist, he had better make up his mind that he has mistaken his vocation, turn his back upon it, go into the woods and live in a hollow tree. The fact is he must give a warm grasp of the hand when he feels like biting off the ear for physical offenses such as shock the senses of sight and smell. The dentist, in short, must expect varicose veins, corns, hemorrhoidal tumors, spinal irritation, headache and heart scaldings as part of his lot in life, and yet go on and endure it cheerfully.

Dr. McCollum desired, if not out of order, to express the sympathy of the Society with Dr. Watt on the peculiar class of his patients.

Dr. Watt replied that he had no reason to complain on that score at all. He was very nicely situated, and had a veiy nice class of patients.

Dr. J. Taft thought Dr. Watt magnified the difficulties. He believed there were a good many good people in the world, and that goodness cropped out where least expected. No one, however, has everything. "Why," he asked, "was it that this Society did not number three times as many, and numbered so few of the profession from Southern Ohio and Kentucky?" Simply because they would not avail themselves of the advantages around them. They are responsible for not doing so, because it is incumbent upon them to avail themselves of every advantage to help them to properly and rightly perform that which they profess to do.

Dr. C. M. Wright remarked that, in his view, the expectations of the dentist could be summed up as follows:

First—To wait ten years for a living business.

Second—To work hard every day.

Third—To be insulted by your patients, for being young till you are old, and then being old, be insulted for that.

Fourth—To have difficulty in collecting your bills.

Fifth--To be an angel of patience, goodness, and earnestness during life.

Sixth—To die poor at an advanced age, and leave his family a small insurance policy.

Dr. Watt desired now to look at the question from the standpoint of the people, and what they had a right to expect in, of and from the dentist. They had a right to expect him to be thoroughly posted in his profession, in its literature, and in the progress he makes in the Dental Association. He should be so qualified in his undertaking the care of human health that no one could question him.

There is still another reason for high expectations from the dentist. The medical profession has given up the care of the teeth and committed the charge of them to the dentist. In view of this the people have a right to expect him to be posted in the highest knowledge. He should know the constitution of the patient, the quality of food best adapted to him, and the mode of treatment proper to his case. He should know the acid that destroys the teeth of the child, and should be able to tell the cause and apply the remedy. He should know the acid and its source, and it is a reasonable requirement of the dentist to act efficiently on that knowledge, or wanting it to make use of every laudable effort to acquire it, by inquiry, by reading, by experiment, and not to rest until he is able to detect the source and apply the remedy, for until dentists can do this, no matter what they call themselves, they will, in reality, be nothing more than mechanical dentists. When they have such knowledge, then and not till then will they merit nor should they bear the honorable title of dental surgeons.

Dr. Wm. H. Morgan, of Nashville, was long persuaded that practice in operative dentistry needed a looking back into first causes. Who is the enemy, and where did he originate? Instead, however, of seeking a definite and specific answer we look at him from the exterior; build a barricade, and fight him over it. If our practice was based upon hygienics, then Dr. Watt's view is right, but the trouble is our patients come to us diseased, and we must combat the enemy as we find him. What avail hygienics when the tooth is decayed? They then only become assistants to mechanical treatment. Then there is a limit beyond which we need not go in looking back. We need not desire to know whether the patient has a French cook or not, so his food is wholesome, but we must look far enough to ascertain that, and it will be well enough to understand the habits of the patient sufficiently to know that they are not positively injurious and detrimental to the teeth.

Dr. A. O. Rawls, of Connersville, Ind., believed that the dentist is governed a good deal by localities. Cincinnati required a different treatment from any city South. He had been South and discovered their mode of treatment to be entirely different from what it is North. They practice after the old, worn out and abandoned methods. They use soft foil almost exclusively in the South, and if they only know it will not do anything as it is done in the North. The question hinges upon what is the requirement for all? Gold foil is universally acknowledged as the best filling for the salvation of the teeth, but here is the difference in the form used North and South just pointed out.

He apprehended, however, that there was a general ignorance on the subject which would never be removed until ignorant men were driven from the profession, or were lifted to the enlightened standard. In Ohio the operation of the law will undoubtedly effect this, and he believed that such legislation in every State will place dentistry on solid ground, and make it all in the eyes of the intelligent world that its high claims demand.

Dr. H. J. McKellops, of St. Louis, did not think it in the scope of any one man's life to embrace all the requirements named by the gentlemen preceeding him, as for himself he only claimed to be a dental surgeon, still he expected something from the patient. He could not go as far as Dr. Watt in saying that the dentist must find causes. If we could only know all about those and how to prevent them, what would be the necessity for dentistry?

He thought the profession forgot or omitted to teach patients to take proper care of their teeth and mouths. There should be a law to compel boarding-school keepers to enforce a care of the teeth on the pupils. The moment we can teach the patient how to take proper care of his mouth, then we reach the perfection of dentistry.

He believed that great benefit resulted to the dentist from the Association. He was free and happy to say that he learned more from attending this Society in one year than in reading all the publications issued on the subject of dentistry. Not that these were to be disregarded, but they had not the vivifying and lasting effect of personal experience and attrition.

He regarded it as a duty to buy all the best appliances. It always doubly repaid him to do so.

He emphasized the importance of having patients come at stated and frequent periods to have their teeth examined, and to keep constantly in their view the necessity for a personal and careful attention to the teeth and mouth.

Subject 2d.—What are the obstacles in the way of a complete fulfillment of professional responsibility, and how are they to be overcome?

Discussion opened by Dr. J. Taft. He thought one great difficulty was in not working up to the highest capacity. The dentist should do every day the very best he could. He had to observe that there is a great variety of opinion as to what a full dental education is, and what constitutes it. There are many men in the profession who totally ignore the many things that go to make up such an education. There are few in it who set a just appreciation upon the facilities surrounding them, and this want of appreciation shows a disregard of them. The dental associations are disregarded. Many enter them merely for the sake of the prestige they think membership will give, yet so really beneficial are they that he did not believe there was any one in the profession who would not be benefited by attending them, while there are many who could be lastingly benefited by taking a course or two in any of the dental colleges. That brings up the question of dental edu cation, a question broad enough to require discussion in and for itself. But in a word, sufficient importance is not attached to collegiate education. The great advantage of it seldom enters the head of the majority, and that is the reason the dental colleges pine, and dwindle, and struggle for existence.

How many in the profession have a proper appreciation for the responsibilities they assume? Very few indeed have as much appreciation as the sculptor who goes to work with chisel and mallet on a block of marble, which he fashions just as he pleases; yet here the dentist takes charge of living organs, of the health and perhaps the life of his patient. There is then great necessity for a deep sense of responsibility and a just appreciation of the duties and the requirements of the profession.

Do we help one another? We meet once or twice a year and talk over our business; we stimulate and encourage one another, and we feel the stronger for it, but there are many men in the profession who shut themselves up in their shells and you don't know where they live, or where to find them, let alone what they do. Fortunately, this class is thinning out, and we hope to live to see them all gone or changed.

Well, we benefit ourselves by giving and are benefited in turn. The best interests of the profession are subserved in this way. To be sure we sometimes run across men who question all the time, who never advance anything themselves, but there is a way to remedy that sort of thing. The true way is to manifest an active living interest and cultivate a sympathetic and reciprocal spirit, then will we build up ourselves and our profession in usefulness and strength.

Dr. Morgan thought Dr. Taft had not touched sufficiently one branch of the subject—dental education. There are 15,000 dentists in the United States. The average working life of the dentist is ten years. The ratio number of students is 2,000, but instead of that number, the 15,000 only send 400

or so annually to the colleges. This is a lamentable condition of affairs, and shows a disregard on the part of the profession for dental education.

Men in the profession should give their fellows the benefit of their thought. It may not seem valuable in their own eyes, but it may and frequently does prove of incalculable value to others.

Dr. Morgan next alluded to the difficulties met with in want of public appreciation, and in the indifference of dentists as a class to each other. He pointed out the importance and value of helping each other by consultation in cases, as in medical practice, and suggested the subject as worthy of present and future consideration

Dr. McCollum desired to express his approbation and hearty concurrence with the remarks of Drs. Taft and Morgan, and regarded the subject of dental consultation as highly important and of the very primest value.

Dr. H. R. Smith was struck with the truthfulness of the allusions to dental education. He was sorry to see that the matriculation fee was more looked for than mental qualification. As a general thing the papers of dental students were miserable. Their language was bad, and many of them could not even spell correctly. Many of them never saw a work on anatomy before entering the college. This state of affairs should not exist. There was no excuse for it. The fact is the food should be chewed a little for them before giving them tough beef. Money is a great want, and it is one the profession is not ready to supply the poor student, as the theological schools sometimes do. He concluded that in dentistry we must go back to the alphabet before creditable graduates can be turned out.

Dr. Taft was attracted by the thoughts expressed by Dr. Morgan, but the question was, how shall the obstacles be removed?

He thought some of them were disappearing. He apprehended that a nearer approach to the medical profession was essential. How? Simply by putting ourselves in position to command its respect, and this the faithful student can do without difficulty. Dr. Taft then instanced a case which a reputable physician referred to him because it properly belonged to the dentist and not to the physician. The influence of such an act on the mind of the patient was of incalculable benefit to the dentist, and this should be the case all the time. It really remained with the dental profession to have it so.

The medical profession should be met on friendly terms; not obtrusively, but in a spirit of appreciation for all that is interesting to it. The medical profession recognizes that our profession has made discoveries, and valuable ones, and that it exceeds it in some things; but this is not enough. We must march with it and keep pace with it for our own benefit, and for the benefit of those who entrust their health and lives to our care.

Dr. James Taylor thought the only way to meet the wants indicated lay through the dental college. He regarded the medical as sympathetic with the dental profession, and altogether willing to recognize it as a specialty, and believed that in twenty or thirty years hence it will be regarded everywhere as such.

Dr. W. P. Horton, of Cleveland, Ohio, thought that the difficulty dated far back. The old practitioners were not educated men. They had good desires and purposes, but lacked discipline and culture. The first he ever saw of dentistry was a medical student plugging teeth by crowding pieces of gold between the teeth.

The lawyers and doctors of forty years ago were learned men, but not so the dentists. There were some dental practitioners then but no profession. The idea was that you could take a young man from the plow and make a dentist of him in three weeks for $25. The venerable Strickland differed materially from this view, for he held that the preparatives of a good education and medical reading were necessary, and after these at least three years' study. Yet, strange as it may appear, the former notion prevails to a large extent at the present time.

Now the non-attendance at the public gatherings of the profession is a great drawback. There are 500 dentists in Ohio, and only fifty or sixty here. Where are they?

Voice—In their shells.

This disdain to assemble and exchange ideas is pitiful. There are 40 dentists in Cleveland, yet not half a dozen can take each other by the hand as brethren. The really good dentists can be counted on your fingers. Where are all the rest? Practicing a trade. They are not gentlemen, not professional men, but a lot of fellows following dentistry as a business; not responsible, not educated, not respected.

Another drawback is the want of appreciation on the part of the public. The majority will go where work is cheap, and there are always to be found cheap fellows who will furnish cheap work. He knew wealthy people who drove in splendid equipages and lived in splendor, yet who would higgle about the price of a piece of dental work, and instanced the case of a wealthy man who called him a swindler for charging an average of less than $4 a piece for gold plugs. This all grew out of ignorance on the part of the people, and the profession was partly to blame for it.

Dr. E. Osmond, of Cincinnati, called attention to one matter that in his estimation required it but had been overlooked. He meant those dental slaughter houses where men mutilated the human form by extracting teeth by aid of laughing gas They never paused to consider if the teeth could be saved by filling, but administered the gas, dug out the teeth, and invited the victim to come again and have his teeth extracted without pain. He was surprised too to find medical men endorsing these butchers who advertised themselves in the newspapers and were ranked by the people as dentists.

He considered that the operative branch of dentistry was injured and the people imposed upon by these fellows, and thought, therefore, that reputable dentists should keep anesthetics on hand for extreme cases, so as to prevent or at least counteract the evil doings of these establishments in destroying the people's teeth.

Dr. Knapp had something favorable to say about the progress of the profession. Dr. Branard, of Chicago, a few years ago complimented the specialty very highly, and observed to him that dentistry had made more progress in twenty years than surgery had in 300.

Dr. Knapp had found as many lame ducks and quacks in medicine as he ever did in dentistry, and upon mature reflection he really felt proud of his profession as a dentist. He would be ashamed now of the work he did only ten years ago. He thought with the progress made and making there was no insuperable difficulty in the way of a recognition on a professional basis by the medical profession.

"What obstacles are in the way of a complete fulfillment of professional responsibility, and how are they to be overcome?"

Dr. W. F. Morrill read the following paper on this subject:

In the every day routine of dental practice there are obviously many hindrances to a complete fulfillment of our responsibility which are worthy of earnest consideration. Progress and development of art and science in dentistry have not yet reached the Utopia of perfection, nor the realization of our hopes and ambition. Impediments still lie along our pathway of duty and of success. We confess with humility that disappointment and baffled skill and failures are too often our experiences. Something like these too often occur, which interfere most grievously with the sacred trusts committed to our care. Valedictorians and retiring presidents of dental societies often admonish us and the classes of students whom they address of the grave responsibilities assumed by becoming dentists; but to the obstacles which visit so seriously the professional career they usually make but a faint allusion.

And now, let us inquire into this responsibility. What is it? It implies trust, guardianship, obligation, bounden duty. Applied to a dental practitioner, it implies that he is intrusted with guardianship of the human teeth, that he is to protect, preserve, and rescue them from harm, disease, and destruction. And that he shall, also, be capable of performing such other necessary operations on the oral cavity as shall contribute to the comfort, happiness, and beauty of mankind. This is the true mission of the dentist. In confiding this trust to him, it presupposes a season of preparation and of culture. The hand must receive a proper amount of training, the eye must be quick to discern and detect, and the mind treasured with the fundamental principles of practice. If he be deficient in any of these essential particulars, he can not feel that weight of responsibility attached to his calling, as one who has devoted his best energies and gifts to the highest attainments possible to be received. Moral obligations rest easily upon the consciences of those who bring but indifferent skill to their aid. Their professional responsibility has but a few obstacles in the way of a complete fulfilment. But, with the most acceptable guardians of the teeth, there is a sensitiveness of feeling and duty, a pride and honor, which multiplies the hindrances to professional obligations.

At an earlier day in our professional career the nature and character of dental operations were far less numerous, and not so complicated as they are now. They embraced only a certain class. Mechanical ingenuity alone seemed to be relied on exclusively for their execution. In fact, this faculty was regarded and is now to some extent, as the *sine qua non* to make a "good dentist." That mechanical skill is a great auxiliary in becoming a dentist must be conceded; that it should receive no disparagement at our hands is equally true; but that this gift must wholly be depended upon, to the neglect of other acquirements, is a wrong apprehension that seriously impairs the advantages which would secure a higher fulfillment of responsibility, must be frankly admitted. Too often the geniuses of our profession squander much time in their laboratories whittling toys, or constructing amusing devices, which should be devoted to the more substantial pursuits of knowledge. The system of education of our dental schools is far in advance of what it ever was before. Our dental literature constantly opens up new avenues of thought, and if we be diligent in appropriating the knowledge here proffered, the obstacles which defeat us in the line of duty must diminish. We are still athirst for knowledge. Ignorance is our greatest obstacle. We are still floundering around, repeating the unanswered questions, who will tell us some infallible method of preserving pulps of teeth when exposed? Who understands with an exact certainty the pathological conditions of each case, and the proper remedies to be prescribed? How many times each year do the "good angels" enlighten us with regard to those methods of filling teeth which are the best and only ones to save them? These are seemingly, to a vast number of the profession, puzzling questions which have not been settled or agreed upon, save among those who dwell in effulgent glory and light. They are embarrassments that clog our professional career. Nor can we expect to make ourselves acceptable guardians, clothed with responsibility, until these impediments are removed. Moreover, on the other hand, we have to contend with much in the conduct of patients which seriously interferes in bringing about desired results. He may not be sufficiently impressed with our efforts to save his teeth; he may not respond to our endeavors; his conduct may be restless, uneasy, and beyond control. Dental operations made under such circumstances are blasted fruits. Earnest appeals, sharp, incisive words, sometimes snatch victory out of the jaws of death, but they can be made available only in certain cases. In my judgment no greater obstacles can be named than the eagerness and frequency of change in our methods of practice, and the adoption of many new appliances of little or no merit. New innovations in dental practice, when the operator is succeeding passably well, should be slow of acceptation and adoption. But a conservatism that admits of no change, always in the same old ruts, more fossilized than the Egyptian mummies, is alike pernicious and reprehensible. For a rational reform be always ready, and abandon that which seemeth not good.

If time and your patience permitted, I might branch out into all the minor details of practice, and show what are my experiences which hinder a complete fulfillment of duty; but I prefer in this not too concise a manner to treat this question, leaving much for your own natural suggestion to add. Wherein I have not stated, I will now hint, how we may overcome many of the obstacles lying in our pathway of duty.

Every meeting of this Society, every meeting of any dental society intelligently conducted, every periodical published in our interest, every college professor and peer in thorough-bred competency, is daily lessening the obstacles with which we have to contend. If we would make ourselves more useful and less ornamental, we should be more industrious in gathering important facts, and bring them here for investigation and profit. How much would our difficulties be overcome if this were done. If we were only faithful and studious to employ our tact and talent in bringing to light and acquaintance the discoveries so often made and announced, we should not be groping along in the narrow way as unacceptable guardians of the teeth; but with a bold, confident step we would be striding along down the broad, sunny avenue of palms where no hindrances obstruct the view, and where professional obligations will be more faithfully accomplished.

SUBJECT 4TH.—What is the present status of the profession in ability to protect, rescue and save the natural teeth?

SUBJECT 5TH.—What are the means requisite for securing the largest measure of success in the preservation of the teeth?

These subjects were taken up for discussion together.

Dr. J. Taft addressed some remarks to the fourth proposition. He said it involved Hygiene. Care must be taken of the teeth before disease sets in, and the probability is that we are too careless in this respect. He would tell mothers to give their children proper food and to look after the general health. He thought, however, that the outlook is becoming larger.

Great care was exercised by the old successful dentists in the matter of hygiene, and that fact alone should instruct us. The time was when we only advised patients to keep their teeth clean, but the best dentists now give strict attention to hygiene and to the general health.

We should have constantly in view the ultimate preservation of the teeth. We see operations every day that will only last a few years at most, and that in performing them the grand object of permanent preservation was not in view. Now, the old operators gave attention to this, and the result is that operations performed twenty and thirty years ago are good to-day and likely to last the lives of the owners.

Much, however, depends upon the patient. When he is away from the dentist he alone can preserve his teeth, but it is the double duty of the dentist to do the work for endurance, and to teach the patient to preserve it.

Dr. Morgan conceived the importance of preserving the temporary teeth for the organization and development and future perfection of the permanent organs. He considered it absolutely necessary to care for the temporary teeth, and regarded the chisel and file as important agents in the work.

Dr. Osmond gave some experiences of practice in the case of the temporary teeth. which were listened to attentively.

Dr. Watt addressed the following remarks on diet to the subjects under discussion:

Dr. Watt said that any eruptive disease during dentition is a great misfortune. Have we not all seen the sad effects of measles, scarlet fever, etc., on the permanent teeth? We can do much good by teaching parents their duty in reference to those diseases. Some are in the habit of exposing children to measles, especially while young, regardless of the state of development of the dental organs. If a child has not had measles by the close of its fifth year, it is very important to avoid the disease till after the fourteenth; and we should carefully point out to parents the reason for this precaution. I have often awakened the attention of parents by telling them the age at which their child had suffered from an eruptive disease, having gained all my information from an examination of the teeth. Having thus secured their attention, we can readily instruct them.

In defending the dental organs against the attacks to which they are liable, it is important to know in each case who is the enemy, and what his mode of warfare. The commander of a fort would not adopt the same plan of defense when attacked by a red man of the forest as when a British regular is his enemy. So we, when we examine the mouth and the general constitution of our patient, should be able to devise a line of defense adapted to the mode of attack. For example, when we examine a mouth and find the mucous membrane flabby and relaxed, the buccal fluid ropy, "oily," or tenacious, in short, when the proportion of mucous is greatly in excess, the local danger to the teeth is the action of nitric acid, resulting in white decay. Now, if we fill the carious cavities without correcting this state of affairs, unless the patient uses the utmost care, and often when he does, we find decay goes on around the borders of the plug. Ignorance of what is duty in these cases, or indifference in regard to it, often results in failure, and a consequent discredit to our profession, even when the manipulations may have been up to the highest style of our art. A patient in the condition described should live mainly on a farinaceous diet. The food should be rich in carbon, but not at all rich in nitrogen. Of course, then, lean meats, eggs, cheese, etc., are to be avoided.

Let us examine another mouth and we may find the membrane nearly normal in appearance, the fluids of the mouth perfectly liquid. If, on applying test paper, we find an acid reaction, in most cases the acid is the hydrochloric. Dissolve a little of this saliva in pure water, even if it is neutral, and add a few drops of its solution to a solution of nitrate of silver. If any soluble chlorides are present there will be a whitish precipitate. In this case the danger is not from nitric, but from hydrochloric acid, and the caries, when it appears, will not be the white, but a variety in which the earthy matter of the tooth is dissolved out, while the organic, in great measure, remains. A patient in such condition should not indulge in salt foods to any great extent, but should very freely use fresh vegetables, milk, etc.

When a patient comes to us fat and flabby (of a lymphatic temperament) rather gouty or rheumatic, and especially if he uses tobacco, we may expect his buccal fluids to contain lactic acid. This acid dissolves, with about equal facility, the animal and earthy constituents of the tooth, producing what is commonly called "chemical abrasion." It makes clean work as it goes; and if you don't believe it, examine my eye-teeth. This acid is often found in the mouths of chronic tobacco chewers. Such a patient should mainly abstain from milk, cheese, sugar, and vegetables containing it, in short, from all substances likely to take on the lactic acid fermentation. He should indulge in lean meats, brown bread, cracked wheat, etc.

There is yet another variety of caries known as the black decay; and it is produced by the action of sulphuric acid. When the breath is loaded with sulphuretted hydrogen (or hydro sulphuric acid, if you like the name better) you may expect to find this variety of dental caries. This is the least disastrous of the four varieties. The tendency to it is to be overcome by avoiding the use of food containing sulphur. Onions, eggs, beefsteak, etc., should be but sparingly indulged in.

Now, I have been able only to give a general hint in this direction; but I hope we will reach something practical in regard to the education of ourselves and our patrons in the care of the teeth.

Dr. Keely thought one reason for the old fillings lasting so long was that the old dentists had better subjects. The trouble now-a-days was the very general soft substance of teeth. He was of opinion, however, that the best fillings made now would endure as long as the old fillings so loudly praised.

Dr. McKellops was of opinion that too much could not be said on the subject of hygiene, but even that well looked after the dentist had to counteract the bad effects of the patients, neglect. If the patient can be given something to remove or arrest decay when it sets in, that will be the first step towards saving the permanent teeth. He would recommend Dr. Arthur's excellent little book to every dentist as an infallible guide.

Dr. Knapp said that all the tendencies of our civilization are against the dentist; the diet and habits of the people especially. If we succeed we must commence with individuals and go back to the very birth, and sometimes back of that, of the child before substantial, satisfactory, and generally good results can be expected.

Drs. McCollum and Baxter took the same ground, the former heartily endorsing Dr. Watt, and the latter amplifying the remarks of Dr. Knapp.

Dr. Horton regarded it as next to impossible to prevent disease and decay in the teeth. The question with him then was how to save them. He thought we should study as Dr. Watt had studied, original sin. The operators of to-day must meet and try to overcome the difficulty as they find it. Many grown people are little better than children in matters of hygiene, and this fact in view reminded him of a very successful physician he knew who practiced forty years without giving any medicine more harmful in its nature than bread pills and colored sugar water. He gave attention solely to dietetics, but if we were to attempt this sort of thing now, he supposed we would be crucified.

Dr. McCollum wanted it distinctly understood that he was a regular candidate for crucifixion.

Dr. Corydon Palmer was much interested in the remarks on constitutional treatment. It seemed to him that the course was onward. Progress has been rapid, yet we have been retarded a good deal by clogs in our own ranks. They came in various shapes, the chisel and file for the mutilation of the teeth being none of the least among them. Then there were the various forms of gold, all differing from the plain smooth foil as it comes from the beater's skins, which the mistaken ideas of the day consider necessary in the way of preparations. He was very much surprised to hear Dr. Osmond recommending nitrate of silver for sensitive teeth. That was the old English mode, which is discarded by the best dentists now.

It seemed to him that true progress did not lie in the way of the variously prepared forms of foil, and believed for substantial reasons that any other than the plain smooth foil already referred to would fall short of the highest and best results. Every sensible dentist must know that fillings made with soft foil will always remain soft. He would then ask why the modes of the past are dragged along and made part of the progress of the day?

Subject 6th.—What is the present status of the profession in ability to supplement the loss of the natural teeth, and the defects occasioned thereby; and what are the impediments in the way of higher attainments in this direction?

Dr. J. Taft alluded first to the improvements made from time to time, and especially to the rapid development of dentistry as an art. The beauty and symmetry of the face, the restoration and preservation of the original form, were all conserved by modern attainments and the aim of the highest art in the profession is to restore not only natural appearance but the distinctness of utterance and enunciation. All this requires skill and study, so that in the construction of substitutes the natural teeth may not be missed to any remarkable external extent. Then again artificial teeth should so perfectly simulate the natural dentals as to secure thorough and easy mastication of the food, but this very important feature is overlooked by the makers of cheap sets. Hasty work will not do. This turning out of teeth by the half bushel a day is not the thing. It will not because it can not give perfect satisfaction. The utmost pains must be taken to adapt the set to each particular individual or the work will be incomplete, and, therefore, unsatisfactory. How often do we see all natural utterance of speech and facial expression ruined by miserable makeshift, cheap artificial work? The very best artificial work is a poor substitute for the natural teeth, and the profession should, therefore, hasten the time when the dreadful necessity for it shall hardly exist, but when that necessity does occur let us be prepared to meet it, not with makeshifts and shams, but with the best possible substitutes, from which alone any degree of satisfaction can reasonably be expected or rendered.

Dr. C. M. Wright spoke emphatically against the use of block teeth as a difficulty in the way of the highest attainments of the art. He was clearly of opinion that no good dentist would use them, or encourage their use in any way.

Dr. Horton considered that a great difficulty lay in the want of a proper appreciation on the part of the people for the best work. They consent to wear cheap work and will go around and hunt it up. The wealthy were no exception to the rule, and, in fact, were more likely to disregard good work just on account of price than those in very moderate circumstances. He knew wealthy people to go from dentist to dentist looking for a cheap set of teeth, just as they would go from one shoe store to another looking for a fit, but he was happy to say that in the end these people all found out that their cheap sets were miserable failures.

Dr. Smith, President of the Society, apprehended that a great difficulty was in the want of a thorough appreciation of the individuality of patients. There was not sufficient care taken to adapt teeth to the complexion, conformation of the face, etc., and each case should have special attention to its own individual wants and necessities, without which, in fact, all attempts would be lame bungling and unsatisfactory both to the patient and to the dentist.

Dr. J. Taft addressed some remarks to the last clause of the subject "What are the impediments," etc. He was clearly of opinion that many could be found in the low estimate that people place on their natural teeth; in the disregard and neglect shown when decay first sets in. They think, and indeed say, that when the teeth are gone they can easily be replaced with a new set, and this is peculiar to that class of people who go shopping to see where they can buy the cheapest. He apprehended that if the facilities for supplying these cheap people with cheap teeth were less, they would be more careful of the natural teeth, and more careful in the selection of substitutes and the makers of them. It was curious to observe that the same carelessness did not exist about the other organs. If a man were to go around manifesting a recklessness about his eye or his nose, simply because he could buy a cheap artificial one, when the natural one was gone, he would be regarded as insane.

The difficulty then is to be met by drawing a line between the true artist and the mere tooth maker. When this is done the people will soon find out the difference, and be educated up to the true, and, therefore, to the high standard. In the meantime the good dentist must draw the line. He must not descend, and he must educate his patients to see the matter in the proper light, and to understand the practical advantage to themselves in avoiding the false and adhering to the true. Dr. Palmer had done much in this direction, and he deserved great credit for it, and would undoubtedly derive advantage from it, but more in the profession must follow his example.

The young man in the profession who desires and aims for this excellence will find strong men to help him, but if he stays down on the low level on the plea of making a living and doing the wrong thing against his better knowledge and in the face of the best light, he will never rise, but sink lower and lower in the scale, and finally drop into scorn and obscurity.

